# Design of Inner Matching Three-Stage High-Power Doherty Power Amplifier Based on GaN HEMT Model

**DOI:** 10.3390/mi15030388

**Published:** 2024-03-13

**Authors:** Renyi Li, Chen Ge, Chenwei Liang, Shichang Zhong

**Affiliations:** Nanjing Electronic Devices Institute, Nanjing 210016, Chinagech_seu@163.com (C.G.); liangcw1008@163.com (C.L.)

**Keywords:** GaN HEMT, EE_HEMT model, Doherty PA

## Abstract

This paper introduces the structure and characteristics of an internal-matching high-power Doherty power amplifier based on GaN HEMT devices with 0.25 μm process platforms from the Nanjing Electronic Devices Institute. Through parameter extraction and load-pull testing of the model transistor, an EE_HEMT model for the 1.2 mm gate-width GaN HEMT device was established. This model serves as the foundation for designing a high-power three-stage Doherty power amplifier. The amplifier achieved a saturated power gain exceeding 9 dB in continuous wave mode, with a saturated power output of 49.7 dBm. The drain efficiency was greater than 65% at 2.6 GHz. At 9 dB power back-off point, corresponding to an output power of 40.5 dBm, the drain efficiency remained above 55%. The performance of the amplifier remains consistent within the 2.55–2.62 GHz frequency range. The measured power, efficiency, and gain of the designed Doherty power amplifier align closely with the simulation results based on the EE_HEMT model, validating the accuracy of the established model. Furthermore, the in-band matching design reduces the size and weight of the amplifier. The amplifier maintains normal operation even after high and low-temperature testing, demonstrating its reliability. In conjunction with DPD (digital pre-distortion) for the modulated signal test, the amplifier exhibits extremely high linearity (ACLR < −50.93 dBc). This Doherty power amplifier holds potential applications in modern wireless communication scenarios.

## 1. Introduction

**Figure 1:**
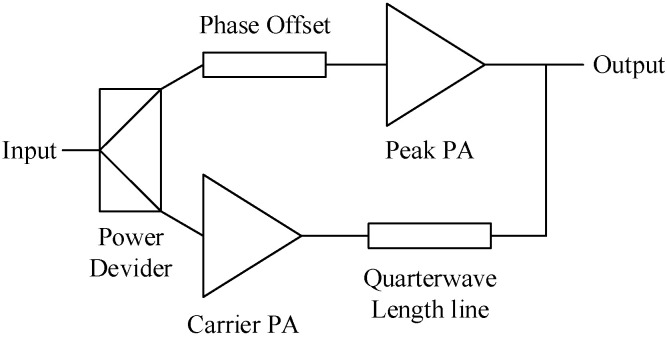
Schematic diagram of traditional Doherty amplifier structure.

Fifth-generation communication technology (5G) adopts sophisticated modulation techniques such as QAM (quadrature amplitude modulation) and OFDM (orthogonal frequency division multiplexing), resulting in a high peak-to-average power ratio (PAPR) in its signal waveform [1,2]. Doherty power amplifiers can maintain high efficiency both in saturation and power back-off, making them suitable for handling 5G communication signals [3]. The schematic diagram of the traditional Doherty architecture is shown in Figure 1. The quarter-wavelength line after the auxiliary amplifier achieves load modulation performance through impedance transformation. The introduction of a 90-degree phase difference at the input ensures phase alignment between the two amplifiers. However, this architecture can only achieve a power back-off range of about 6 dB, which falls short of meeting the requirements for processing signals with high PAPR.

**Figure 2:**
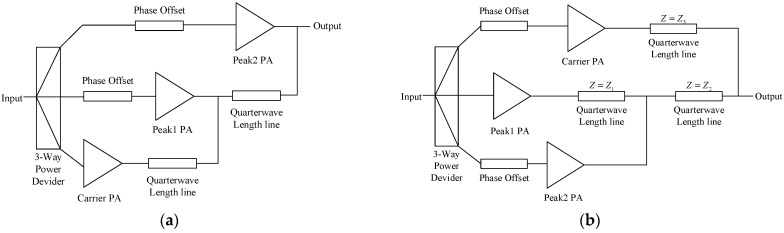
Schematic diagrams of two three-stage Doherty power amplifier structures, (**a**) is extension of the traditional Doherty structure and (**b**) is an improved structure proposed by NXP.

Asymmetric architectures, as illustrated in the literature [4,5,6,7,8,9,10,11,12,13,14], have improved the traditional Doherty power amplifier structure to enhance its power back-off range. The auxiliary amplifier, compared with the main amplifier, has a higher saturated output power, achieving a power back-off range of approximately 7–8 dB. Additionally, two three-stage Doherty structures, as shown in Figure 2a,b, can achieve power back-off ranges greater than 8 dB. The three-stage Doherty architecture in Figure 2a is an extension of the traditional Doherty structure. The carrier amplifier and the first peak amplifier together form a two-stage Doherty power amplifier, which then combines with the second peak amplifier to form a new Doherty power amplifier.

In 2011, NXP proposed an improved three-stage Doherty power amplifier structure [15], as shown in Figure 2b, which has been widely adopted, as documented in references [16,17,18,19,20]. Compared with the architecture shown in Figure 2a, this design effectively improves the gain compression of the main amplifier and further extends the power back-off range. The load modulation behavior of this architecture is analyzed later. 

On the other hand, GaN HEMT (gallium nitride high-electron-mobility transistor) devices, due to their broad frequency response and high power density, are widely used in communication, radar, aerospace, and other fields [21,22,23,24,25]. The accuracy of HEMT device models is crucial for MMIC (microwave monolithic integrated circuit) design success and performance. The EE_HEMT model proposed by Agilent Technologies in 1993 is a widely used nonlinear compact model for HEMT devices, providing accurate characterization of the RF properties of the device. Additionally, the EE_HEMT model is a compact model that maintains high accuracy even in scaled applications. In this paper, an EE_HEMT scalable model for a GaN HEMT device with small gate width was established through DC scanning testing, S-parameter testing, and extraction of intrinsic and parasitic parameters. The model was further validated through load-pull testing and simulation, confirming its accuracy in large-signal conditions. The results of testing on the actual circuit indicated close agreement between simulation and measurement, with simulation errors of less than 5% for drain efficiency and less than 1 dB for gain under large-signal excitation, validating the precision of the established EE_HEMT model.

## 2. Materials and Methods

### 2.1. Introduction of GaN HEMT Devices

**Figure 3:**
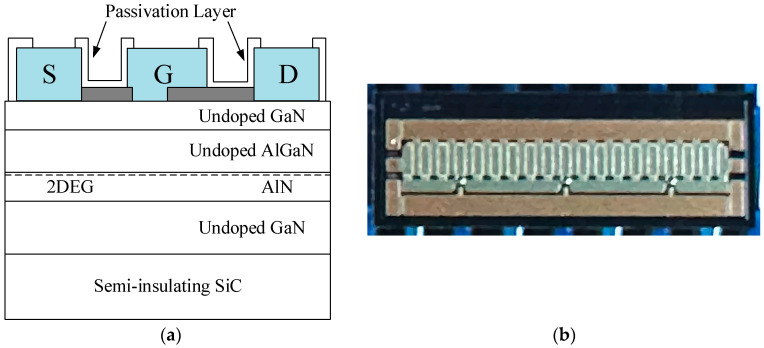
Schematic diagram of the longitudinal structure of GaN HEMT (**a**) and photograph of (**b**) its use in this design.

This design utilizes a 7.2 mm gate-width GaN HEMT device developed by Nanjing Electronic Devices Institute. Under a bias condition with a drain–source voltage (*V_DS_*) of 28 V, the device can provide a continuous wave saturated output power density exceeding 4.5 W/mm [26]. Its longitudinal structure, as shown in Figure 3a, includes a SiC substrate, an undoped GaN buffer layer, an AlN insertion layer, and an AlGaN barrier layer. The SiC substrate serves the purposes of support, heat dissipation, and electromagnetic shielding. The AlN insertion layer enhances electron mobility to improve high-frequency characteristics and concurrently reduces scattering effects caused by lattice mismatch. The addition of a gate field plate helps lower the peak electric field, thereby increasing the breakdown voltage of the device. Simultaneously, to minimize thermal resistance and enhance reliability, the SiC substrate is thinned and gold-plated on the reverse, improving heat dissipation capabilities. In addition, the SiN passivation layer covering the entire device surface can effectively enhance the transistor’s radiation resistance capability [27]. The entire chip is grounded through vias to minimize parasitic capacitance and inductance, thereby enhancing the high-frequency characteristics of the device. Figure 3b shows a photograph of the GaN HEMT used in this design. The device has a single-finger gate width of 200 µm and a total gate width of 7.2 mm. The overall size of the entire chip is 2.62 mm × 0.93 mm.

### 2.2. Parameter Extraction and Model Establishment

**Figure 4:**
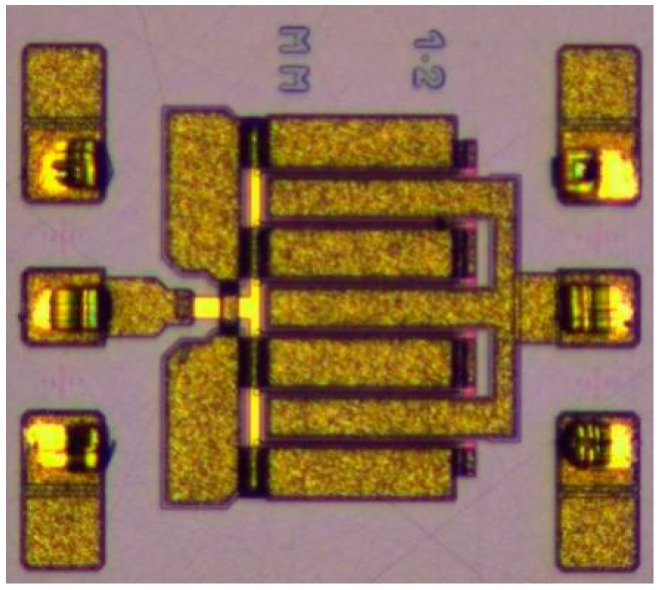
Microscopic photo of 1.2 mm gate-width model transistor.

The EE_HEMT model was established based on a 1.2 mm gate-width model transistor with the same process technology as the GaN HEMT in this design. It has a single-finger gate width of 200 µm, with a total of 6 gate fingers, resulting in an overall gate width of 1.2 mm. Figure 4 shows a microscopic photograph of this model transistor.

**Figure 5:**
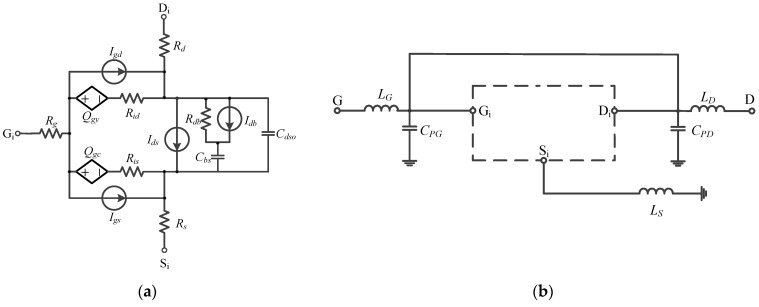
Intrinsic equivalent circuit diagram (**a**) and parasitic parameter equivalent circuit diagram (**b**) of the large-signal EE_HEMT model.

DC scanning testing and S-parameter scanning testing in the frequency range of 0.4 GHz to 35 GHz with a pulse period of 1 ms and a duty cycle of 10% were conducted to extract intrinsic and parasitic parameters of the model transistor described above. By fitting the intrinsic and parasitic parameters, an equivalent circuit for the large-signal EE_HEMT model, as depicted in Figure 5a,b, was established. The extracted intrinsic parameters of the transistor were *R_G_* = 978 mΩ, *R_D_* = 833 mΩ, *R_S_* = 133 mΩ, and the parasitic parameters were *L_G_* = 118 pH, *L_D_* = 111 pH, *L_S_* = 10.6 pH, *C_PG_* = 31.5 fF, *C_PD_* = 39.4 fF.

**Figure 6:**
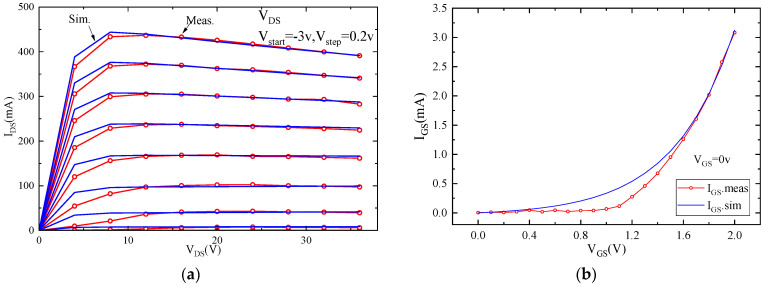
Comparison between simulation and measurement of DC characteristics of 1.2 mm gate-width model transistor, (**a**) drain current and (**b**) gain current.

**Figure 7:**
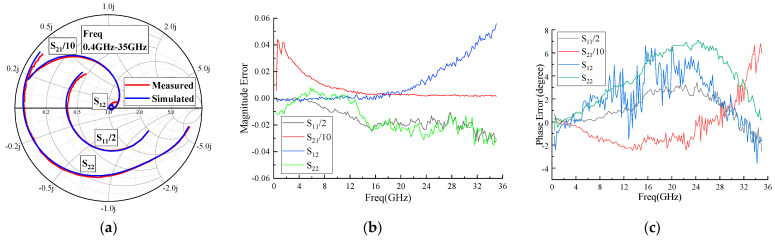
Comparison between simulation and measurement of 0.4–35 GHz S-parameters of 1.2 mm gate-width model transistor (**a**), magnitude error (**b**) and phase error (**c**).

**Figure 8:**
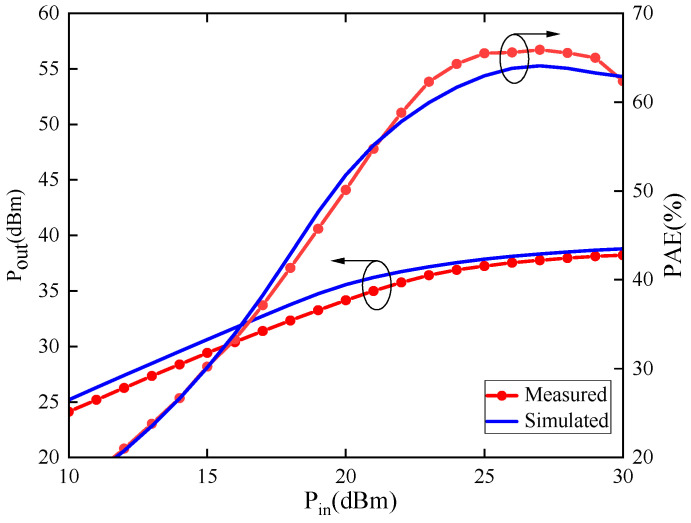
Comparison between simulation and measurement of output power and PAE of 1.2 mm gate-width model transistor.

Additionally, load-pull testing was conducted on the model transistor to obtain information such as maximum saturated output power and the highest PAE, to validate the accuracy of the model under large-signal conditions. Figure 6 and Figure 7 represent the DC characteristics and S-parameter test results compared with the model simulation results. In Figure 6a,b, the DC simulation and test results for drain current and gate current are presented, respectively. Figure 7b,c show the magnitude and phase errors of the S-parameters within the frequency range of 0.4–35 GHz. The magnitude error is less than ±0.05, while the phase error is distributed within the frequency range of −5 degrees to +8 degrees. Figure 8 displays the test results for load-pull output power and PAE, along with the simulation results. The results indicate a close agreement between simulation data and measured data, confirming the accuracy of the model structure and parameters.

### 2.3. Theory and Circuit

#### 2.3.1. Load Modulation Behavior Analysis of Three-Stage Doherty PA

The Doherty PA structure used in this study is shown in Figure 2b; it is assumed that it is composed of symmetric devices and all cells saturate simultaneously at maximum input power. The relationship between the output and back-off power for the three-stage Doherty PA is derived as follows:

*P*_*out*,*max*_ = 3/2·*V_DC_*·*I_MAX_* (1)

*P*_*out*,*k*2_ = 1/2·*V_DC_*·*I*_*C*,*k*2_ (2)

*P*_*out*,*k*1_ = 1/2·*V_DC_*·(*I*_*C*,*k*1_ + *I*_*P*1,*k*1_) (3)

where

*I*_*C*,*k*1_ = 2/3·*k*_1_·*P*_*out*,*max*_/*V_DC_* (4)

*I*_*P*1,*k*1_ = 2/3·(*k*_1_ − *k*_2_)/(1 − *k*_2_)·*P*_*out*,*max*_/*V_DC_* (5)

*I*_*C*,*k*2_ = 2/3·*k*_2_·*P*_*out*,*max*_/*V_DC_* (6)

The back-off output power can be expressed in the following form:

*P*_*out*,*k*1_ = *k*_1_^2^·*P*_*out*,*max*_ (7)

*P*_*out*,*k*2_ = *k*_2_^2^·*P*_*out*,*max*_ (8)

Combining the equations above, we can determine the values of *k*_1_ and *k*_2_:

*k*_1_ = 1/3, *k*_2_ = 1/2 or 1/3 (9)

**Figure 9:**
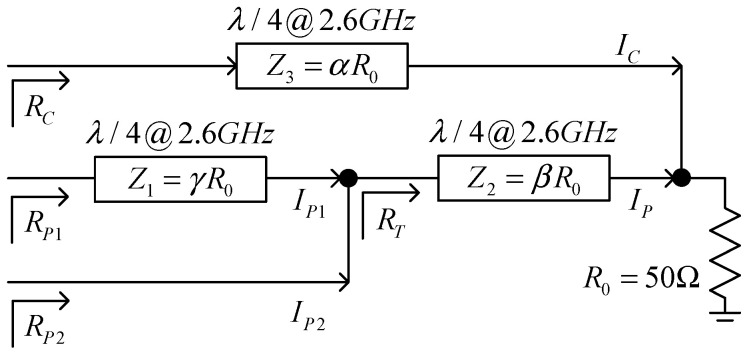
Schematic diagram of power-combining network.

Under different biases, the three-stage Doherty PA can have two or three maximum efficiency points. Figure 9 shows the structure of the power-combining network, and according to the principles of dynamic load modulation, the characteristic impedances of the three microstrip lines can all be calculated.

The fundamental drain current ratio between the main amplifier and the peak amplifier can be defined as follows:

*δ*_2_ = *I_P_*_2_(*v_in_*)/*I_P_*_1_(*v_in_*) (10)

*δ*_1_ = (*I_P_*_2_(*v_in_*) _+_ *I_P_*_1_(*v_in_*))/*I_C_*(*v_in_*) (11)

Table 1 shows the variation in *δ*_1_ and *δ*_2_ as *v_in_* increases.

Assuming the load impedance *R*_0_ is 50 Ω, and defining the characteristic impedances of each quarter-wavelength line as *Z*_3_, *Z*_2_, and *Z*_1_ for each section as *αR*_0_, *βR*_0_, and *γR*_0_, respectively, the load impedance for each amplifier can be derived as follows:

*R_C_* = *α*^2^·*R*_0_/(1 + *δ*_1_) (12)

*R_T_* = *β*^2^·*R*_0_·*δ*_1_/(1 + *δ*_1_) (13)

*R_P_*_1_ = *γ*^2^·*R*_0_/*β*^2^·(1 + *δ*_1_)/(*δ*_1_·(1 + *δ*_2_)) (14)

*R_P_*_1_ = *β*^2^·*R*_0_^2^·(δ_1_·(1 + *δ*_2_))/(*δ*_2_·(1 + *δ*_1_)) (15)

Combining with Table 1, the load impedance for each amplifier can be derived as follows:

*R_C_* = *α*^2^·*R*_0_    *v_in_*/*v_ma__x_* = 1/3    = 1/2·α^2^·*R*_0_   *v_in_*/*v_max_* = 2/3   = 1/3·*α*^2^·*R*_0_     *v_in_*/*v_max_* = 1 (16)

*R_P_*_1_ = ∞        *v_in_*/*v_max_* = 1/3   = 3·*γ*^2^·*R*_0_/*β*^2^    *v_in_*/*v_max_* = 2/3   = 3/4·*γ*^2^·*R*_0_/*β*^2^   *v_in_*/*v_max_* = 1 (17)

*R_P_*_2_ = ∞        *v_in_*/*v_max_* = 1/3   = ∞          *v_in_*/*v_max_* = 2/3   = 4/3·*β*^2^·*R*_0_     *v_in_*/*v_max_* = 1 (18)

If each amplifier is matched to 50 Ω when *v_in_*/*v_max_* = 1, then according to the above equations, *Z*_1_, *Z*_2_, and *Z*_3_ can be calculated as follows:

*Z*_3_ = *α*·*R*_0_ = Sqrt(3)·*R*_0_ (19)

*Z*_2_ = β·*R*_0_ = Sqrt(3/4)·*R*_0_ (20)

*Z*_1_ = *γ*·*R*_0_ = 1·*R*_0_ (21)

#### 2.3.2. Circuit Design

Load-pull simulation was performed on the transistor with a gate width of 7.2 mm, and the results indicate maximum output power of 46.2 dBm and peak PAE of 75.4%. Striking a balance between power and efficiency, the chosen saturation output impedance of the chip is 5.2 + j × 5.5 Ω. At this point, the output power is 46 dBm, and the PAE is 69.4%, meeting the design requirements.

The output matching employs an L-C-L matching structure implemented through microstrip lines, effectively controlling the circuit’s second harmonic. The second harmonic impedance is maximized to make the amplifier operate in class-F^−1^ mode, thereby enhancing the efficiency of the single amplifier.

**Figure 10:**
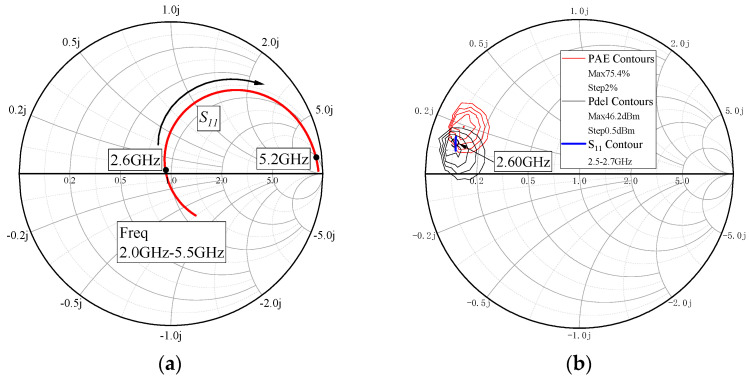
Contour of S_11_ in intrinsic current source plane (**a**) and contour of S_11_ for output-matching network (**b**).

The contour of S_11_ in the intrinsic current source plane on the Smith chart is depicted in Figure 10a, and the contour of S_11_ for output matching network is depicted on the Smith chart in Figure 10b.

**Figure 11:**
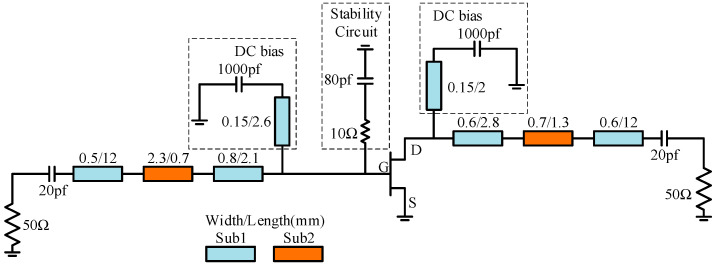
Schematic diagram of single amplifier.

Through source-pull simulation, input impedance of the transistor was determined to 0.3 − j × 2.2 Ω. The input matching network structure is similar to the output matching network structure, with a relatively simple design that can match input impedance to 50 Ω. Additionally, a parallel-connected capacitor-resistor stability circuit was added at the input of the transistor to ensure the stable operation of the amplifier. Figure 11 shows the schematic diagram of a single amplifier.

**Figure 12:**
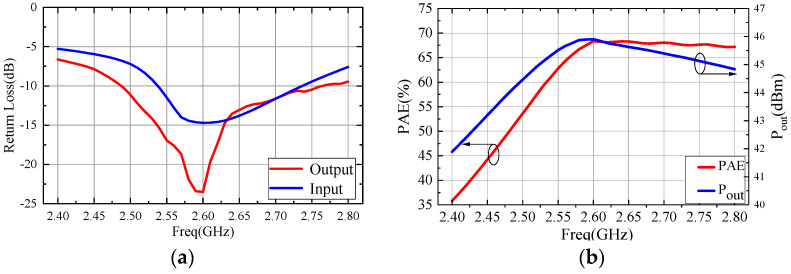
Simulation results of single amplifier (**a**) return loss and (**b**) output power and PAE.

A frequency sweep large-signal simulation was performed on the designed single amplifier under bias conditions of *V_GS_* = 28 V and *V_DS_* = −2.1 V, with an input power of 34 dBm. The simulation results are presented in Figure 12. Figure 12a displays the large-signal simulation results of input and output return loss and Figure 12b displays the output power and PAE. The simulation results indicate that the saturated output power and PAE of the single amplifier were 45.9 dBm and 68.2%, respectively. These values closely align with the load-pull data, confirming the accuracy of the circuit design. Furthermore, due to the introduction of a grounded circuit, the simulation of the stability factor (K) for the single amplifier within the operating frequency band was consistently greater than 1. This indicates that the amplifier can operate stably within the frequency range, ensuring its stability.

In order to reduce the circuit size, the practical microstrip circuit was manufactured on an alumina ceramic substrate with a dielectric constant of 9.9 and thickness of 380 µm, as well as barium titanate ceramic substrates with dielectric constant of 85 and thickness of 180 µm. The microstrip circuit and the chip are sintered onto a molybdenum-copper alloy carrier, and they are interconnected through wire bonding. Additionally, 20 pF chip capacitors were added at the input and output positions of the amplifier to isolate the DC component. Furthermore, 1000 pF chip capacitors were also introduced to filter out high-frequency noise at the feeding point. The amplifier has dimensions of 19.2 mm × 10 mm and is securely fastened to the test fixture using screws.

**Figure 13:**
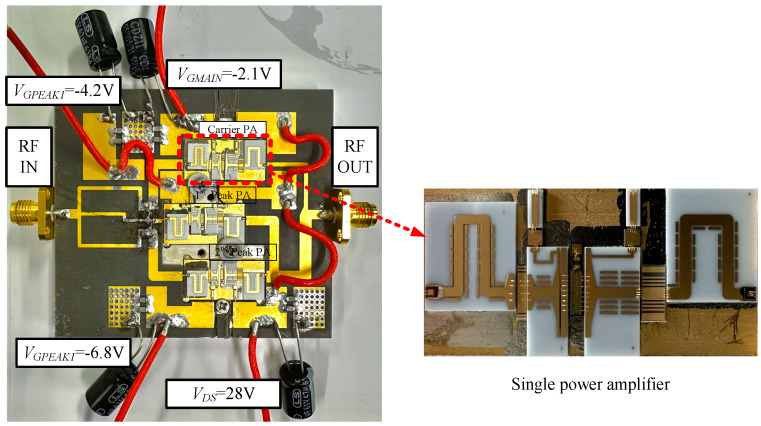
Photograph of whole Doherty PA and single PA.

The input power divider of the Doherty power amplifier adopts a three-way Wilkinson power divider structure with equal power splitting, and the impedance of each port of the power divider is 50 Ω. Additionally, to maintain the phase consistency of the three power amplifiers, 90° phase compensation lines with characteristic impedance of 50 Ω have been added before the main power amplifier and the second peak power amplifier. The power-combining circuit follows the structure described in Section 2.3.1. Both the power divider and the power-combining circuit have been implemented using microstrip circuits. These circuits were manufactured on PTFE material substrate with low dielectric constant of 2.2 and thickness of 762 μm to ensure that the microstrip widths are sufficiently large to meet the requirements for high power. They are both sintered onto the test fixture and connected to single amplifiers, as described above, using wire bonding. SMA connectors were added at the input and output positions of the power amplifier for test purposes. Properly located pads for feeding and grounding were introduced, and the grounding pads were connected to the test fixture through vias. In addition, capacitors of 100 pF, 1000 pF, and 43 μF were placed between the feeding and grounding pads to filter out high-frequency noise. Figure 13 shows a photograph of the whole Doherty power amplifier. The overall dimensions of the circuit are 66 mm × 60 mm, and its weight is 103.4 g.

## 3. Results

### 3.1. CW Test

As described above, this study employed the Doherty amplifier structure shown in Figure 2b. In this configuration, for each single amplifier, the other two amplifiers act as feedback loops introduced at the input. The introduction of feedback has a significant impact on the Doherty amplifier when a single amplifier experiences self-oscillation, leading to a substantial increase in noise and severe interference with the integrity of the signal. To avoid such occurrences, it is crucial to ensure the stability of the Doherty amplifier.

In Section 2.3.2, the stability of the single amplifier was verified through simulation, and prior to the continuous wave testing of the Doherty amplifier, its stability was experimentally confirmed. Under bias conditions of *V_DS_* = 28 V, *V_GMAIN_* = −2.1 V, *V_GPEAK_*_1_ = −4.2 V, and *V_GPEAK_*_2_ = −6.8 V, with no RF signal input, the drain current was zero. As the gate voltage of the main amplifier gradually increased, the drain current also steadily and linearly increased. This observation indicates that the Doherty amplifier does not undergo self-oscillation and can operate stably.

**Figure 14:**
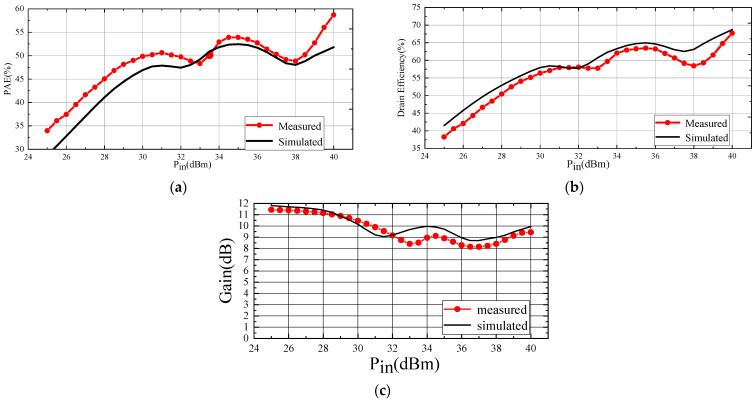
CW test results and simulation results of PAE (**a**), drain efficiency (**b**), and gain (**c**).

After verifying the stability of the Doherty amplifier under the same bias conditions, continuous-mode power scanning test and frequency scanning test were conducted on the designed Doherty power amplifier. Figure 14a–c depict the results of the power scanning tests; Figure 14a,b show the test results and simulation results for PAE and drain efficiency, while Figure 14c illustrates the gain characteristics of the Doherty power amplifier.

As shown in Figure 14, at 2.6 GHz, the saturated output power was 49.7 dBm, with a drain efficiency exceeding 67% and gain exceeding 9 dB. At the output power of 40.5 dBm, corresponding to a 9 dB power back-off, the drain efficiency remained above 55%, and the gain was above 8 dB. Comparison with simulation results reveals that, under large-signal excitation, the measured drain efficiency and gain exhibit errors of less than 5% and less than 1 dB, respectively. The overall trend is consistent with the simulation results, confirming the accuracy of the model. The sources of error may originate from two aspects: bonding wires and thermal effects. It is challenging to accurately describe the shape of bonding wires, gold ribbons, and the location of pads during the simulation process. Additionally, the heat generated by the Doherty amplifier can reduce the drain efficiency, and the EE_HEMT model has certain limitations in accurately characterizing the self-heating effects of the transistor. This limitation results in an inability to accurately simulate the thermal effects of the transistor.

We observed that the trends of the curves obtained from simulation and experimentation were similar but not entirely identical. In comparison to the test results, the simulation results show an earlier occurrence of the first maximum efficiency point. This discrepancy arises because the model was established based on parameters extracted in the transistor’s on state, introducing errors in describing the transistor’s deep Class-C-mode operating state. In model-based simulations, the auxiliary amplifier is entirely shut off during Class-C operation, whereas in reality, a small portion of the main amplifier’s power leaks into the auxiliary amplifiers, preventing a complete off state and causing the maximum efficiency point to appear later.

Additionally, according to Figure 14a,b, it is noted that the simulated results for power-added efficiency are slightly lower than the test results, while the relationship of drain efficiency between simulation and test is reversed. The probable cause of this trend is that the actual input signal was smaller than the ideal input signal in the test. The actual input signal, generated by the signal generator and amplified by the solid-state amplifier, was slightly reduced due to the connection losses between devices and the noise from the signal generator to the solid-state amplifier. In contrast, the simulation uses an ideal input signal.

**Figure 15:**
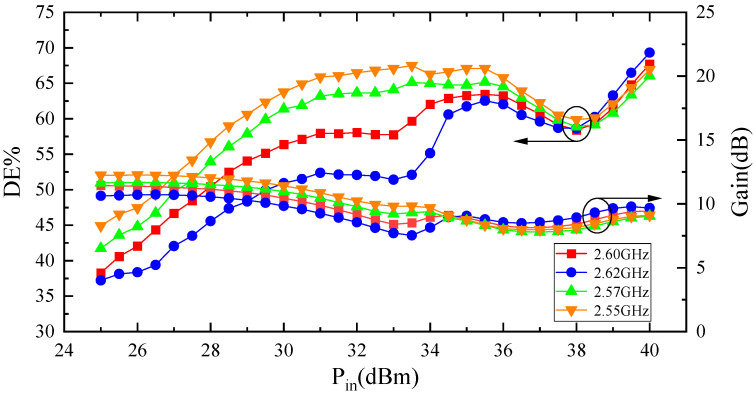
Test results results of drain efficiency and gain at 2.55 GHz–2.62 GHz.

Figure 15 indicates the relationships between the input power and efficiency of the Doherty power amplifier within a frequency range of 2.55 GHz to 2.62 GHz. As shown in the graph, this Doherty power amplifier exhibits stable performance within bandwidth of 70 MHz.

Table 2 presents a comparison between this work and other three-stage Doherty power amplifiers. Compared with other results, this study demonstrates larger saturated output power, higher drain efficiency, and higher linearity.

### 3.2. Modulated Signal Test

Linearity testing was conducted using a modulation signal test system. A Rohde & Schwarz vector signal generator, model SMW200A, was employed to generate the modulation signal, and a spectrum analyzer, model FSW, was used to test the EVM (error vector magnitude) and ACLR (adjacent-channel leakage ratio).

**Figure 16:**
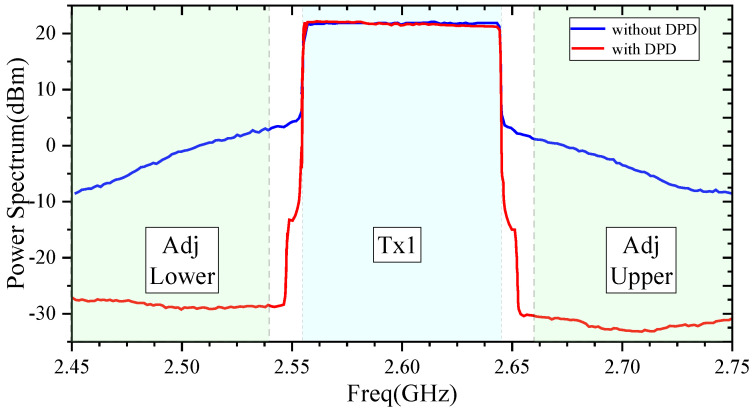
Test results of ACLR with 10 MHz QPSK signal.

The vector signal generator generated a 10 MHz bandwidth QPSK signal with PAPR of approximately 8.3 dB. The amplifier was biased in the same way as in the continuous wave test, with *V_DS_* = 28 V, *V_GMAIN_* = −2.1 V, *V_GPEAK1_* = −4.2 V, and *V_GPEAK2_* = −6.8 V. The test process incorporated DPD technology, and the ACLR test results are depicted in Figure 16. The ACLRs of lower and upper adjacent channels were −22.19 dBc and −23.66 dBc without DPD, and while using DPD, the ACLRs of lower and upper adjacent channels were 50.93 dBc and 52.0 dBc, indicating the high linearity of the Doherty power amplifier.

### 3.3. Temperature Test and Aging Test

Temperature can significantly impact the DC and microwave characteristics of GaN HEMT devices, with performance degradation being more pronounced at high temperatures. The reasons for this are as follows. Firstly, an increase in junction temperature leads to a reduction in the carrier mobility and saturation drift velocity, diminishing the device’s switching speed and subsequently affecting its high-frequency performance. Additionally, in HEMT devices, the gate contact to the semiconductor substrate junction is an Ohmic contact, while the source and drain contacts to the semiconductor substrate are Schottky contacts. Elevated temperatures accelerate diffusion between the metal and semiconductor, degrading both ohmic and Schottky contacts. This results in a reduction in the barrier height, lowering the breakdown voltage and power-handling capacity of the device, which is detrimental to stable device operation. Furthermore, prolonged operation at high temperatures may also impact circuit components. Excessive temperatures can lead to wire bond failures, and the Q-value of chip capacitors may decrease, affecting the overall circuit performance. In summary, under high-temperature conditions, the reliability and electrical performance of GaN HEMT devices and circuits tend to degrade, while the opposite is true under low-temperature conditions.

**Figure 17:**
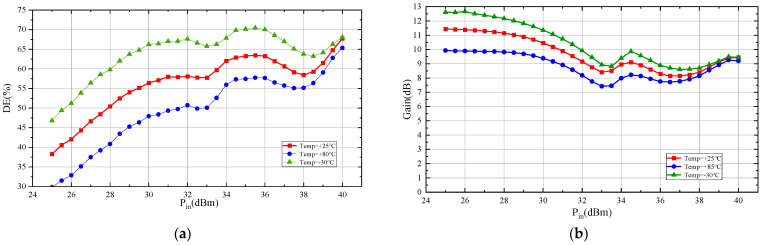
Test results of drain efficiency (**a**) and gain (**b**) at different temperatures.

In order to validate the circuit’s reliability, high-temperature (+85 °C) and low-temperature (−30 °C) tests were conducted on the Doherty power amplifier under the same conditions as the continuous wave test. Figure 17a,b present the drain efficiency and gain results of power scanning tests at high, room, and low temperatures.

As depicted in the figures, the designed Doherty power amplifier operates normally at both +85 °C and −30 °C temperatures. In the power back-off range, its drain efficiency changes by less than ±8%, and gain changes are within ±1.5 dB, showing consistent trends. These results indicate that the Doherty power amplifier exhibits high reliability under different temperature conditions.

In addition, in order to test long-term stable operation capability, aging tests were conducted on the Doherty power amplifier under room-temperature conditions. Under the same bias conditions, the power amplifier worked continuously for 160 h at input power levels of 31 dBm and 40 dBm, corresponding to the back-off and saturated points of the Doherty power amplifier, respectively. The results showed that amplifier could still operate normally after the aging test with almost consistent performance. The designed Doherty power amplifier demonstrates capacity for long-term stable operation, exhibiting a certain degree of reliability.

## 4. Discussion

This study conducted a theoretical analysis of a three-stage Doherty power amplifier power-synthesis network and designed a three-stage high-power Doherty power amplifier based on the theoretical analysis. The test results indicate that the designed Doherty power amplifier can effectively extend the power back-off range of traditional Doherty amplifier architectures, consistent with the theoretical analysis. Additionally, as described in Section 2.3.2, the harmonic matching technique employed in the single amplifier enhances the overall efficiency of the Doherty power amplifier to a certain extent. In comparison to recently reported Doherty power amplifiers, this study demonstrates advantages in performance metrics such as output power and efficiency.

Future research will focus on expanding the operational bandwidth of the Doherty power amplifier while ensuring output power. Bandwidth expansion may be achieved by reducing the use of quarter-wavelength lines to improve the power-combining network and implementing a wideband matching strategy for the single amplifier. These improvements are expected to further enhance the performance of the Doherty power amplifier across different frequency ranges, better meeting the demands of practical applications.

## 5. Conclusions

In summary, we have presented a miniaturized GaN HEMT high-power Doherty power amplifier based on the 0.25 μm process platform of the Nanjing Electronic Devices Institute. We established the EE_HEMT model of the chip through parameter extraction and load-pull testing. Using this model as a foundation, we designed a Doherty power amplifier. Experimental results indicate that at 2.6 GHz under continuous wave test conditions, the saturated output power exceeds 49.7 dBm, drain efficiency surpasses 67%, and gain is over 9 dB. At a 9 dB power back-off point, the drain efficiency remains above 55%. The performance is consistent within the frequency range of 2.55 GHz to 2.62 GHz. Furthermore, the test results align closely with the simulation results based on the model. Under large-signal excitation, the measured drain efficiency error is less than 5%, and the gain error is less than 1 dB, validating the accuracy of the model and circuit design. Additionally, this amplifier features compactness and lightness and exhibits stability at both high and low temperatures. The amplifier holds practical significance for modern wireless communication hardware platforms.

## Figures and Tables

**Table 1:** The values of *δ*_1_ and *δ*_2_ as *v_in_* increases.

*v_in_*/*v_max_*	1/3	2/3	1
*δ*_1_	0	1	2
*δ*_2_	0	0	1

**Table 2:** Compared with other three-stage Doherty PAs.

Units	Architecture	f_0_ (GHz)	BW (MHz)	P_MAX_ (dBm)	DE (%) @PBO	Back-Off Range	ACLR
[6]	2-way	2.43	750	44.6	49	9	−46.2 dBc
[7]	2-way	2.14	10	36.9	55.7	6.5	−25.0 dBc
[16]	3-stage	2.655	15	50.5	55.4	8.5	−40 dBc
[18]	3-stage	0.75	300	46.1	50	12	−50.2 dBc
[19]	3-stage	2.14	100	45.3	55	10	−49.8 dBc
[20]	3-way	2.1	600	46	53	9.5	−30 dBc
This work	3-stage	2.6	70	49.7	57.9	9	−50.93 dBc

## Data Availability

Data are contained within the article.
